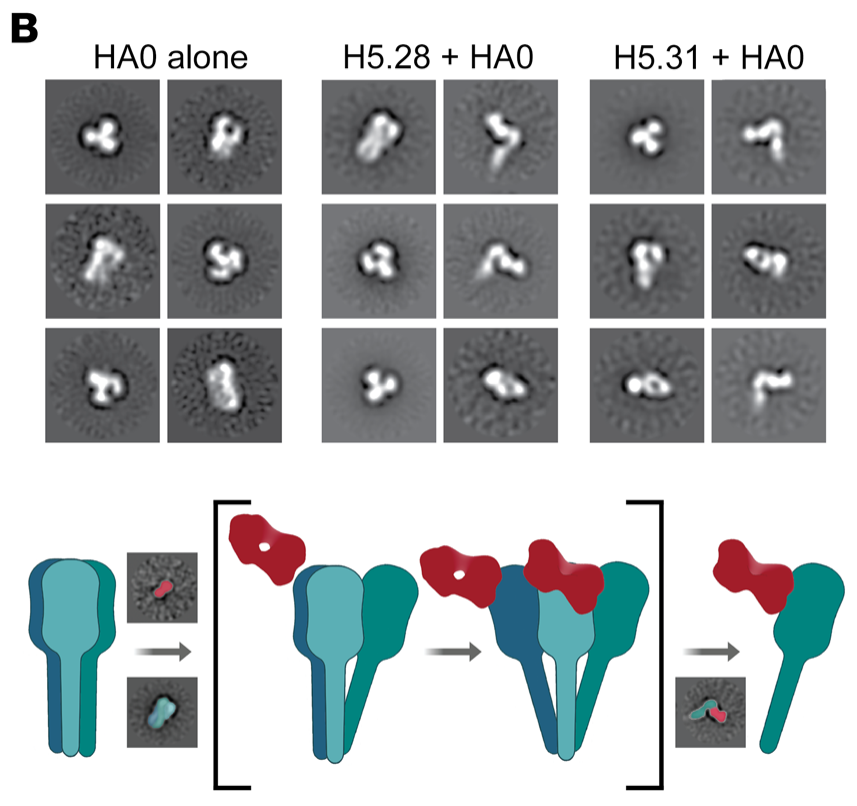# Corrigendum to Canonical features of human antibodies recognizing the influenza hemagglutinin trimer interface

**DOI:** 10.1172/JCI196506

**Published:** 2025-08-01

**Authors:** Seth J. Zost, Jinhui Dong, Iuliia M. Gilchuk, Pavlo Gilchuk, Natalie J. Thornburg, Sandhya Bangaru, Nurgun Kose, Jessica A. Finn, Robin Bombardi, Cinque Soto, Elaine C. Chen, Rachel S. Nargi, Rachel E. Sutton, Ryan P. Irving, Naveenchandra Suryadevara, Jonna B. Westover, Robert H. Carnahan, Hannah L. Turner, Sheng Li, Andrew B. Ward, James E. Crowe

Original citation: *J Clin Invest*. 2021;131(15):e146791. https://doi.org/10.1172/JCI146791

Citation for this corrigendum: *J Clin Invest*. 2025;135(15):e196506. https://doi.org/10.1172/JCI196506

In Figure 2B of the original article, there was an error in the top-left H5.31 + HA0 image, which was an inadvertent duplication of the bottom-right H5.28 + HA0 image. The corrected figure, based on the original source data, is provided below. The HTML and PDF versions of the article have been updated.

The authors regret the error.

## Figures and Tables

**Figure:**